# miR-181a involves in the hippocampus-dependent memory formation via targeting PRKAA1

**DOI:** 10.1038/s41598-017-09095-3

**Published:** 2017-08-16

**Authors:** Sun-fu Zhang, Jun-chen Chen, Jing Zhang, Jian-guo Xu

**Affiliations:** 1 Department of Neurosurgery, West China Hospital, Sichuan University, Chengdu, Sichuan P. R. China; 2 Department of Neurosurgery, The First People’s Hospital of Yibin, Yibin, Sichuan P. R. China; 3 Department of Neurosurgery, Sichuan 81 Rehabilitation Center, Chengdu, Sichuan P. R. China

## Abstract

Post-transcriptional gene regulation by microRNAs (miRNAs) is involved in memory formation. However, the roles of individual miRNAs in these processes remain largely unknown. In this study, we want to clarify the role of miR-181a in hippocampus-dependent memory formation. A transient increase in miR-181a expression was observed after conditioned fear conditioning (CFC) and object location task (OLT) training. Selective overexpression or inhibition of miR-181a in the dorsal hippocampus (DH) via the injection of a miR-181a agomir or antagomir enhanced or impaired the CFC- and OLT-dependent memory formation, respectively. Using bioinformatics and luciferase assays, we identified PRKAA1 as a potential target gene of miR-181a. After CFC or OLT training, the expression and activity of PRKAA1 decreased as miR-181a expression increased and was effectively blocked by the miR-181a antagomir. Moreover, microinjection of the PRKAA1 agonist AICAR or inhibitor compound C in the DH reversed the roles of the miR-181a agomir or antagomir in CFC- and OLT-dependent memory formation. In conclusion, this work provides novel evidence describing the role and mechanism of miR-181a in hippocampus-dependent memory formation, which sheds light on the potential regulation of cognition and future treatments for cognitive disorders.

## Introduction

MicroRNAs (miRNAs) are short (~22 nt), non-coding RNAs that act as translational repressors to regulate the stability of target mRNAs by binding to complementary sequences in the 3′-untranslated regions (3′UTRs) and mediating non-cleavage degradation. Based on 2–8 nt seed region complementarity, the expression of hundreds of predicted targets genes is regulated by a single miRNA, which involves in diverse biological processes and diseases development^1–4^.

According to recent studies, miRNAs participate in neuronal functions, including long-term memory (LTM) formation, synaptic plasticity and dementia^2, 5–9^. Gene expression and synthesis of new protein are crucial for the LTM formation^10, 11^. Therefore, the roles of miRNAs in this process have become clear. Based on emerging evidence, miRNAs, including miR-9–3p, miR-124, miR-132, miR-134, and the miR-183/96/182 cluster, among others, play essential roles in the formation of many kinds of memories^6–8, 12, 13^. In addition to these reported miRNAs, the effects of many miRNAs, which are highly expressed and widely distributed in the brain, on synaptic plasticity, learning and memory are still largely unknown.

miR-181a, a member of the miR-181s family, has been implicated as a regulator of multiple biological processes, such as cell proliferation and invasion^14–17^. The closely relationship between abnormal expression of miR-181a and the clinical outcomes of many kinds of cancers have attracted the attention of many researchers^16, 18–21^. miR-181a is also highly expressed in the hippocampus, a key region involved in the formation of many kinds of memories^22, 23^. In a previous study, miR-181a inhibited the development of hippocampal neurons and synaptic functions by negatively regulating the expression of CREB1 or GluA2, respectively^24, 25^. According to the study by Huang, miR-181a may play a role in impairing the spatial memory of rats with pentylenetetrazol-induced epilepsy^26^. In addition, miR-181 overexpression might be an important factor that contributes to AD neuropathology^27^. All these results indirectly imply that miR-181a may play an important role in hippocampus-dependent memories, but direct evidences is still lacking.

In this study, miR-181a was involved in the formation of contextual fear and object location memories. PRKAA1 was one of the important target genes of miR-181a during the formation of these hippocampus-dependent memories.

## Materials and Methods

### Animals

The animals used in this study were approved by Institutional Animal Care and Treatment Committee of Sichuan University (ACTC). Eight-week-old male C57BL/6 J mice were used for all experiments. Animals were group-housed (3~4 mice per cage) under a 12/12 h light/dark cycle in standard laboratory cages, with food and water ad libitum.

All experiments were performed in accordance with the relevant guidelines and regulations.

### Tissue isolation

The tissue isolation was performed according to previous study^28^. In brief, the dorsal hippocampus (DH) of the mice was defined as anteroposterior (AP): −0.94 to −2.30 mm. The brains of the mice were quickly removed at the desired time points and placed in a mouse brain slicer (RWD). Coronal sections (1 mm thick) were collected, and the DH were isolated under a dissecting microscope following delineations from the mouse brain atlas. The tissues were stored at −80 °C until use.

### Surgery, cannula implantation and microinjection

Mice were deeply anesthetized with a ketamine/xylazine mixture and mounted in a stereotaxic apparatus (8003, RWD Life Science). The eyes of the mice were protected by ophthalmic gel. The 26-gauge guide cannulas (Plastics One) were bilaterally implanted into the DH. The coordinates (in reference to bregma) were as follows: lateral (L), ±1.1 mm; AP, −1.80 mm; dorsoventral (V), and −1.0 mm. The guide cannulas were fixed by dental cement. Then miR-181a agomir, antagomir or their control (20 nM, 0.5 μl/per site respectively, RiboBio.Co. Guangzhou) were injected using a 33-gauge infusion needle (1.0 mm longer than the guide cannulas, Plastics One) connected to a 10 μl Hamilton microsyringe at a rate of 0.1 μl /min. After the injection, the needle was left for an additional 5 min before withdrawal. After surgery, the animals were allowed to recover for 1 week prior to behavior tests. The 5′-prime-AMP-activated protein kinase (AMPK) specific activator 5-aminoimidazole-4-carboxamide-1-β-d-ribofuranoside (AICAR, 0.01, 0.1 or 1 mM, Sigma) and inhibitor compound C (CC, 0.1, 1 or 10 mM, Merck) were injected 30 min before the behavior test via the same infusion needle as above.

### Bioinformatics analysis

To predict the target genes of miR-181a in the mouse hippocampus, a series of bioinformatics analyses were used. Firstly, we predicted the target genes for miR-181a using the following prediction tools: TargetScan (http://www.targetscan.org/) and miRDB (mirdb.org/miRDB/). The preliminary target genes predicted by both the two tools were analyzed further by KEGG analysis. We selected the pathways which were involved in learning and memory. Then we got 20 predicted targets. Among the screened genes of miR-181a, genes with loss or gain of function that induced any changes in LTP or memory in previous reports took priority over other candidates.

### Luciferase assay

Luciferase activity assay was conducted using the Dual-Luciferase® Reporter Assay Kit (Promega) according to the manufacturer’s instructions. HEK293T cells and HT-22 cells (ATCC) were seeded in 96-well plates. 100 ng wild-type or mutant luciferase reporter constructs (termed WT or MUT) were co-transfected into cells with 100 nmol/L miR-181a agomir or 100 nmol/L miR-181a agomir control by using Lipo2000. Luciferase activity assay was conducted 48 hours after transfection using the Dual-Luciferase Assay System. Firefly luciferase activity was normalized to the corresponding Renilla luciferase activity. All experiments were carried out 5 times.

### Behavior paradigms

#### Contextual fear memory (CFC)

The mice were put into a standard fear-conditioning chamber (25 × 25 × 25 cm, Panlab) for conditioning training. Firstly, the mice were allowed to habituate for 120 s without any stimulation (acclimation), then they were given 3 consecutive footshocks (0.7 mA, 1 s duration each) through a stainless steel grid floor. Each foot shock was separated by 60 s interval. After the last shock, mice were left in the chamber for additional 60 s before back to the home cages. One (Short-term memory, STM) or 24 hours after training (LTM), mice were placed back to the previous conditioning chamber where training occurred without foot shock. The times of mice with freezing behavior were recorded during the 5 min test.

### Object location task (OLT)

The experiment was performed as described previously^29^. For habituation, mice were exposed to an open field arena (40 × 40 cm) with 35 cm high walls, for 15 min for 2 d without the objects. Two black, triangle-shaped stickers were placed on the north and east sides of the wall to provide visual cues. During the training session, mice were allowed to explore two identical objects for 5 min. Ten minutes (STM) or 24 hours after training (LTM), one of the objects was moved to a new location (displaced object). Then the mice were placed back to the arena facing the unchanged object and began 5 min exploration, which was defined as the test session. The activity of the mice during training and test sessions were recorded with a digital camera placed above the arena. The time that the mice spent in sniffing or exploring the object within 1 cm of the object was manually counted as the exploration time. The discrimination index was calculated as ([displaced object exploration time/total object exploration time] × 100). All sessions of the task and analysis were performed blindly. During the training session, mice that showed > 65% preference for any object were excluded.

### Open field test

Locomotor activity and anxiety state were measured using an open field test. Each subject was allowed to move freely in the open field apparatus (40 cm × 40 cm × 30 cm). The video tracking system (Smart 3.0) was used to record the distance traveled and time spent in the inner field of mice. The total distance traveled in the arena was recorded to evaluate the locomotor activity. Time spent in inner area was used to show the anxiety state of mice. The inner areas were defined as a square section 10 cm away from each of the walls. Data were collected for 10 min.

### Hot plate test

The hot-plate apparatus consisted of a metal plate kept at 55.0 ± 0.3 °C. Mice were placed on the plate within the confine of a Plexiglas cylinder (15 cm inner diameter). The time of mice to the first hind-paw response was recorded. The hind-paw responses were foot shakes or a paw licks. A cut-off time of 30 s was used to prevent tissue damage.

### RNA extraction and reverse transcription - quantitative real time PCR (RT-qPCR)

Total RNA of mouse hippocampus was isolated using TissueLyser (Qiagen) in Trizol reagent (Invitrogen). Total RNA was extracted by phenol–chloroform precipitation. Purified total RNA (500 ng) was then reversely transcribed using RNase free DNase (Promega) and reverse transcribed using miScript II RT kit (Qiagen). qPCR was performed in a Cycler (Bio-Rad) using SYBR-Green (Roche) with the U6 small nuclear RNA as an internal control. Each sample was assayed in duplicate and the levels of miRNA were normalized for each well to the levels of U6 using the 2^−△△CT^.

### Immunochemistry

Mice were deeply anesthetized with a ketamine/xylazine mixture and perfused with 4% paraformaldehyde (PFA) in PBS. The brains were removed and kept in 4% PFA overnight at 4 °C, followed by equilibration in 30% sucrose before sectioning. Brains were coronal sectioned at a 40 μm thickness with a cryostat (HM 550). Sections were incubated in a blocking solution (0.3% Triton X-100, 0.1% BSA, 10% normal goat serum) for 1 h and then incubated with Alexa Fluor® 488 conjugated avidin (Invitrogen, 1:1000). Sections were then imaged with a fluorescent microscope (IX51; Olympus).

### Western blot

The samples were homogenized in ice-cold RIPA buffer, which contained 25 mM Tris-HCl (pH 7.6), 1% NP-40, 0.1% sodium dodecyl sulfate (SDS), 150 mM NaCl and 1% sodium deoxycholate with phosphatase and protease inhibitors. The homogenized suspension was centrifuged at 14,000 × g for 15 min at 4 °C. The supernatant was collected and its protein level was measured by a BCA protein assay. Equal amounts of protein were subjected to SDS-PAGE with the following antibodies: anti-PRKAA1 antibody (abcam, 1:1000), anti-actin (Cell Signaling, 1:5000), phosphorylated (p-) tuberous sclerosis complex 2 (TSC2, Ser1387, Cell Signaling, 1:1000), acetyl-CoA carboxylase (ACC, Cell Signaling, 1:1000), p-ACC (Ser79, Cell Signaling, 1:1000), p-p70S6K (Thr389, Cell Signaling, 1:1000), p70S6K (Cell Signaling, 1:1000) and TSC2 (Epitomics, 1:1000). The goat anti-mouse or anti-rabbit secondary antibodies (Calbiochem, 1:1000) were used. Immunoreactive bands were visualized by enhanced chemiluminescence (ECL, Pierce). Densitometry analysis on the bands was calculated by Image J.

### Statistics

Data were analyzed by one-way analysis of variance (ANOVA), followed by least significant difference post hoc comparisons. Significance was set at P < 0.05. Results were expressed as mean ± SEM. Data analyses were performed using the SPSS statistical program, version 18.0.

### Data Availability

The datasets generated and analysed during the current study are available from the corresponding author on reasonable request.

## Results

### The expression of miR-181a was transiently increased in the DH after CFC and OLT training

**Figure 1:**
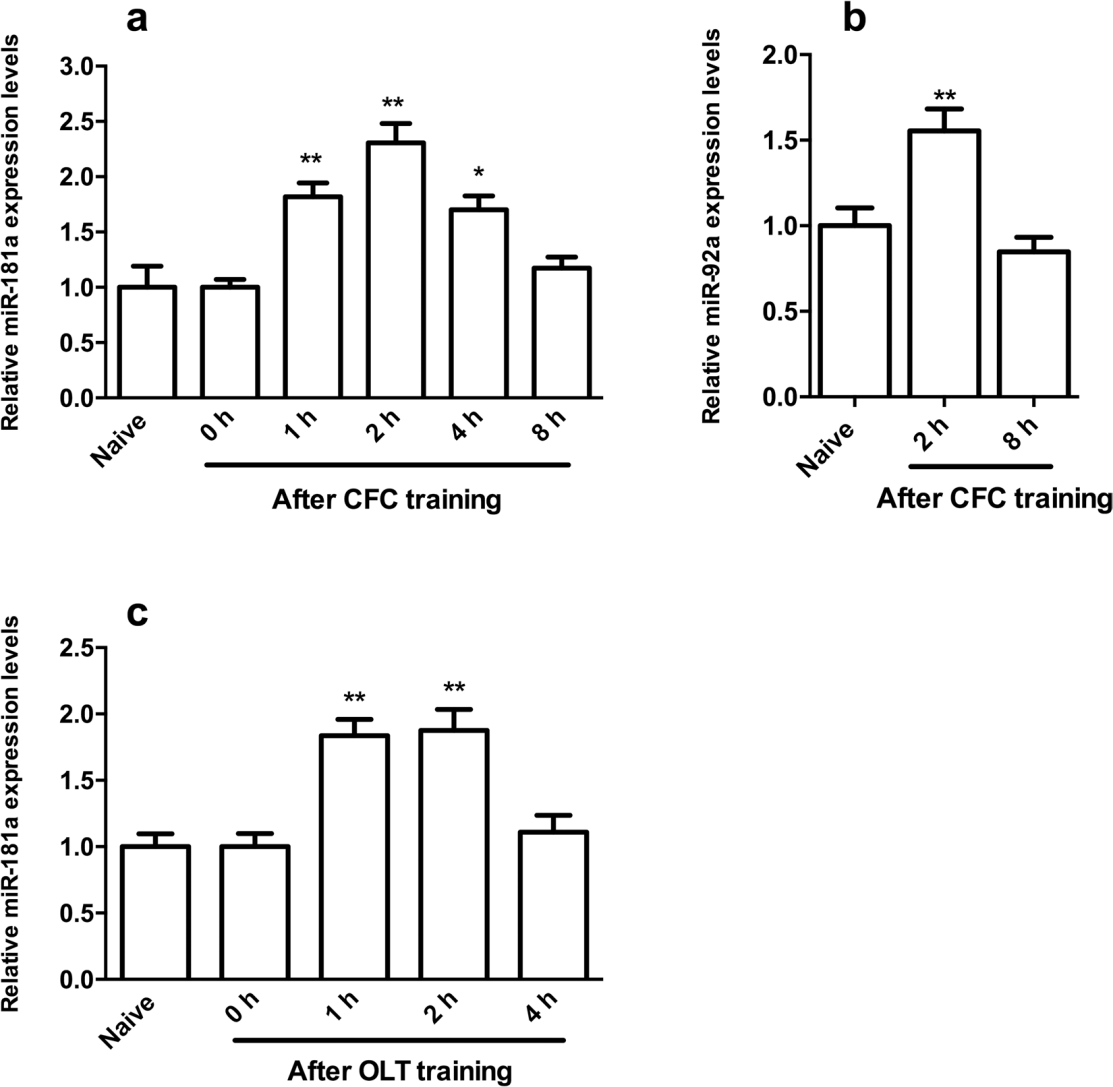
The expression of miR-181a was transiently upregulated in the DH after CFC and OLT training. (**a**) qPCR analysis showing the relative levels of miR-181a expression in the mouse DH at different time points after CFC training. *p < 0.05, **p < 0.01, compared with the naïve group. (**b**) Relative levels of miR-92a expression in the mouse DH 2 h or 8 h after CFC training. **p < 0.01, compared with the naïve group. (**c**) Relative levels of miR-181a expression in the mouse DH at different time points after OLT training. **p < 0.01, compared with the 0 h group. n = 6 per group. All values are presented as means ± SEM.

We first selected the CFC and OLT paradigms and observed the changes in miR-181a expression during the formation of these memories to investigate the roles of miR-181a in hippocampus-dependent memory formation. The expression of miR-181a increased significantly 1–4 h after CFC training and returned to the baseline 8 h later (*p* < 0.01, one-way ANOVA, Fig. 1a). Based on a previous report^30^, we further selected miR-92a as a positive control to detect the changes of its expression in our samples and exclude false positive results. The expression of miR-92a increased 2 h after CFC training and decreased back to the baseline 8 h later (*p* < 0.01, one way ANOVA, Fig. 1b). These results were consistent with the previous report and proved that our experimental system was successfully established. Next, we observed the changes in miR-181a expression after OLT training. The expression of miR-181a also exhibited transient increase from 1 to 2 h after OLT training and returned to the baseline 4 h later (*p* < 0.01, one-way ANOVA, Fig. 1c). Based on these results, miR-181a might be involved in these two hippocampus-dependent memory paradigms.

### Regulation of miR-181a expression in the DH affected LTM formation following CFC and OLT

**Figure 2:**
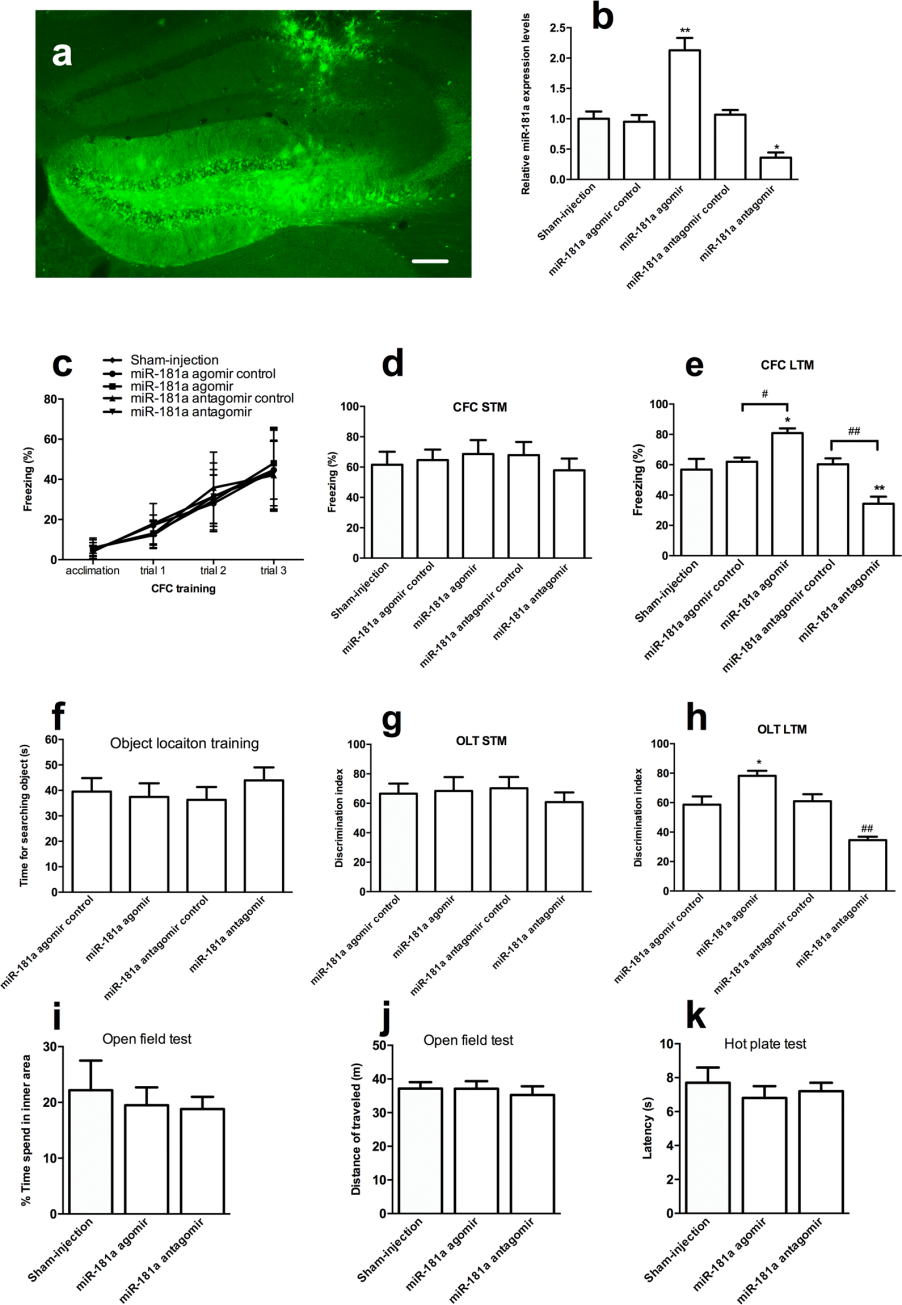
miR-181a was involved in LTM formation following CFC and OLT. (**a**) Representative image showing the location and extent of diffusion of the miR-181a antagomir in the DH. Scale bar, 200 μm. (**b**) Relative levels of miR-181a in the DH after miR-181a agomir or antagomir injection. *p < 0.05, **p < 0.01, compared with the Sham-injected group. n = 5 per group. (**c**) Freezing response during the CFC training. The freezing percentages in the acclimated period (120 s) and each post-shock intervals (60 s per trial) were recorded. (**d–e**) Freezing response 1 h (**d**) and 24 h (e) after CFC training. *p < 0.05, **p < 0.01, compared with the Sham-injected group. ^#^ *p* < 0.05, ^##^ *p* < 0.01, compared with the agomir or antagomir control group, respectively. n = 7 per group (**d**). n = 10 per group (**e**). (**f**) Time spent for searching objects during OLT training. (**g–h**) Discrimination index 10 min (**g**) and 24 h (**h**) after OLT training. *p < 0.05, compared with the miR-181a agomir control group. ^##^ *p* < 0.05 compared with the miR-181a antagomir control group. n = 7 per group (**g**). n = 10 per group (**h**). (**i–j**) Time spent in the inner area (**i**) and total distance traveled in the open field test (**j**). (**k**) Latency in the hot plate test. n = 8 per group. All values are presented as means ± SEM.

We microinjected a miR-181a agomir or antagomir into the DH to enhance or inhibit miR-181a function, respectively, and to further evaluate whether the increased levels of miR-181a functioned during hippocampus-dependent memory formation. One week after the microinjection of the miR-181a agomir or antagomir, the location and diffusion area were examined by localizing the biotin tag on these molecules with avidin–biotin binding methods. The miR-181a agomir or antagomir transfected a large number of hippocampal cells (Fig. 2a). Moreover, the expression of miR-181a in the DH was decreased by more than 60% after the antagomir injection (*p* < 0.05), whereas its expression increased approximately one-fold after the agomir microinjection (*p* < 0.01, Fig. 2b). Based on these results, the miR-181a agomir or antagomir was microinjected into the mouse DH, and the mice were evaluated in the CFC and OLT paradigms one week later. Neither the miR-181a agomir nor antagomir impacted the freezing response during CFC training (*p* > 0.05, one-way ANOVA, Fig. 2c) and 1 h after CFC training (*p* > 0.05, one-way ANOVA, Fig. 2d). However, when mice were tested 24 h after CFC training, the percentage of freezing time increased and decreased in the miR-181a agomir- (*p* < 0.05) or antagomir-injected mice, respectively (*p* < 0.01, Fig. 2e), suggesting that miR-181a was required for the formation of CFC LTM memory.

Moreover, the discrimination index in miR-181a agomir-treated mice increased significantly 24 h after OLT training (*p* < 0.05 Fig. 2h); the opposite result was observed in the miR-181a antagomir-treated mice (*p* < 0.01, Fig. 2h). However, there were no changes in the discrimination index among these groups 10 min after OLT training (*p* > 0.05 Fig. 2g), suggesting that miR-181a was required for the formation of OLT LTM memory.

We performed the open field test and hot plate test to exclude abnormal effects of the miR-181a agomir and antagomir treatments on mice’s anxiety levels, motor skills, and pain sensitivity, respectively. The miR-181a agomir or antagomir injection did not affect the time spent in the inner square (*p* > 0.05, Fig. 2i) and the distance the mice traveled in the open field test (*p* > 0.05, Fig. 2j). Moreover, miR-181a did not change the latency in the hot plate test (*p* > 0.05, Fig. 2k). Thus, the miR-181a agomir or antagomir treatment did not affect the anxiety levers, locomotor activity and pain sensitivity of the mice.

Based on these results, miR-181a was necessary and sufficient for LTM formation following the CFC and OLT.

### PRKAA1 was one of the predicted targets of miR-181a

**Figure 3:**
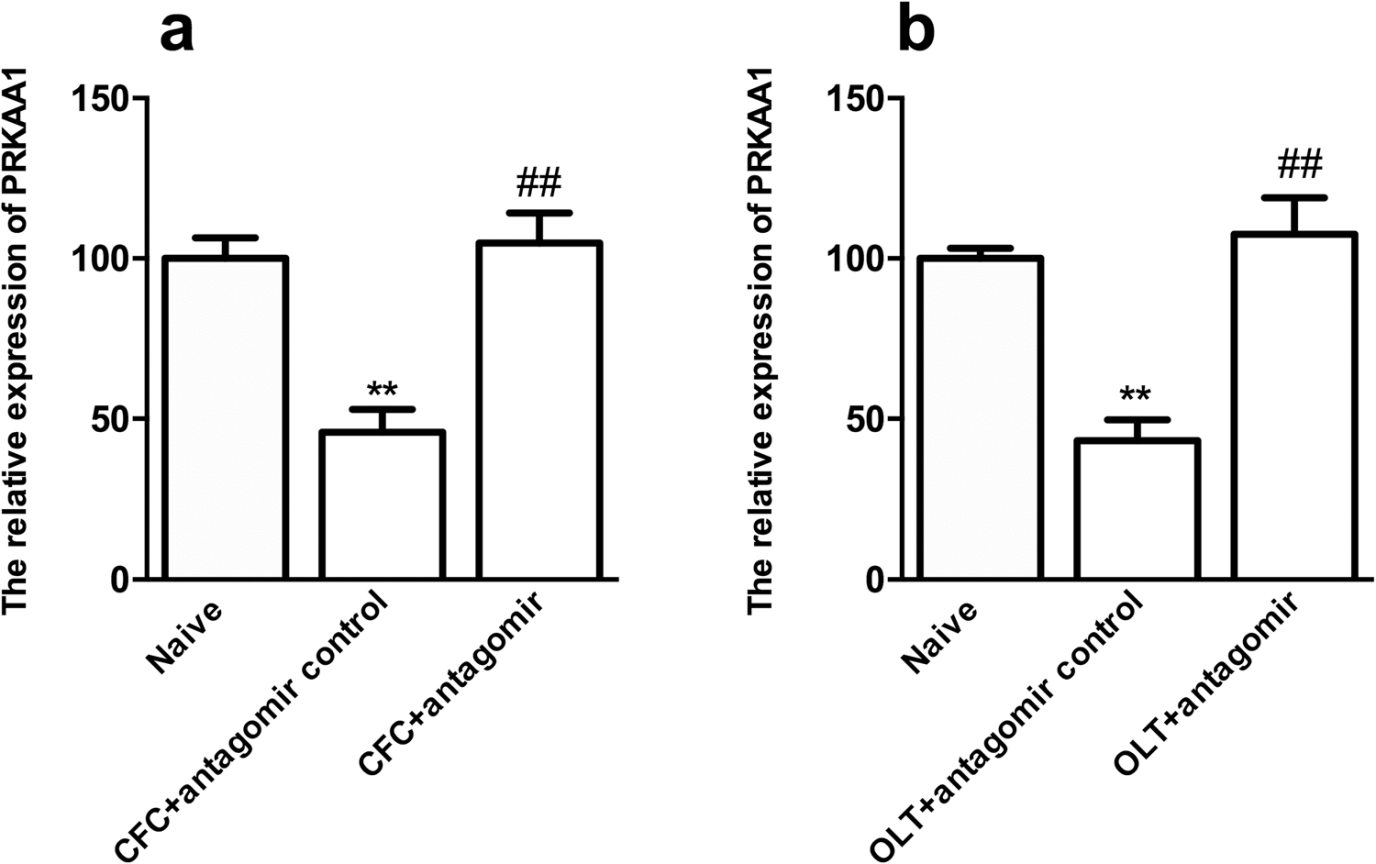
The expression of the PRKAA1 mRNA was regulated by miR-181a during the formation of CFC- and OLT-dependent memories. (**a**) qPCR analysis showing the relative levels of PRKAA1 expression in the mouse DH 2 h after CFC training. **p < 0.01, compared with the naïve group. ^##^ *p* < 0.01, compared with the CFC + antagomir control group. (**b**) Relative levels of PRKAA1 expression in the mouse DH 2 h after OLT training. **p < 0.01, compared with the naïve group. ^##^ *p* < 0.01, compared with the CFC + antagomir control group. n = 5 per group. All values are presented as means ± SEM.

**Figure 4:**
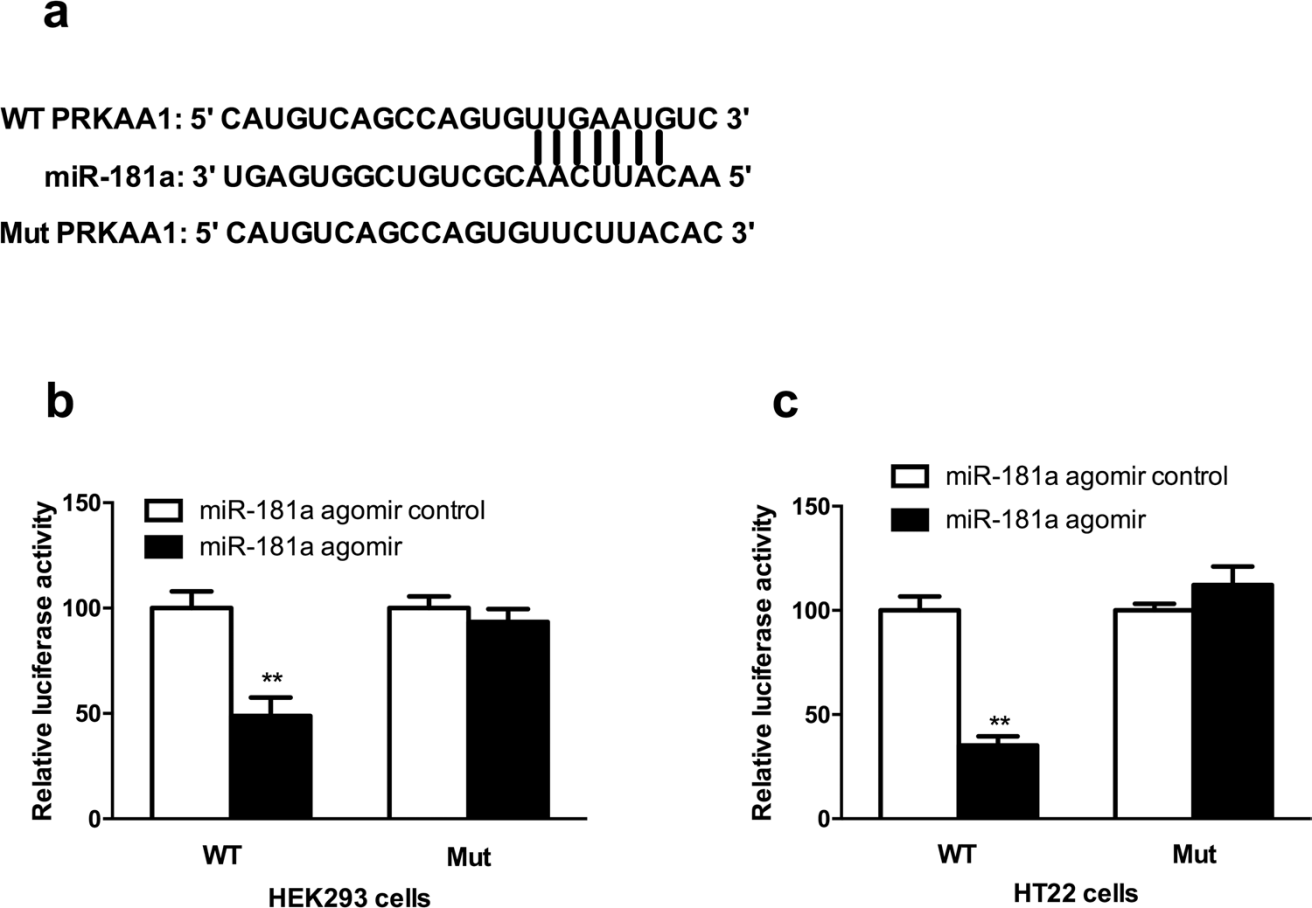
miR-181a mediated PRKAA1 expression by directly binding to its 3′-UTR in HEK293T and HT-22 cell lines. (**a**) Complementary sequences between wild-type (WT) or mutant PRKAA1(Mut) 3′-UTRs in the mRNA and miR-181a. (**b**) HEK293T cells or (**c**) HT-22 cells were co-transfected with the miR-181a agomir or miR-181a agomir control and the luciferase reporter construct containing the WT or Mut PRKAA1 3′-UTR. For each experiment, the results were normalized to the luciferase activity detected in the cells transfected with the miR-181a agomir control and the WT PRKAA1 3′-UTR. **p < 0.01, compared with the WT + miR-181a agomir control group. n = 5 per group. All values are presented as means ± SEM.

Next, we wanted to determine the underlying mechanism by which miR-181a regulates LTM. According to the bioinformatics analysis described in the materials and methods, 20 genes, including MAP2K1, PPP3R1, IRS2, MAP3K3, PRKCD, SOS1, YWHAG, EPHA4, NFAT5, PAK4, PAK7, SEMA4G, DDIT4, PRKAA1, ITGA6, PDGFRA, RRAS2, WASL, NLK and TCF7L2, were predicated as direct targets of miR-181a and might be involved in hippocampus-dependent memory formation. Modification of PRKAA1 expression were previously shown to impair or improve L-LTP^31^. Moreover, the mRNA levels of PRKAA1 decreased significantly 2 h after CFC and OLT training (both p < 0.01) and were reversed by the miR-181a antagomir treatment (*p* < 0.01, Fig. 3a,b). For these reasons, we selected PRKAA1, one of the important molecules in the mTOR signaling pathway, for further studying. A luciferase reporter assay was performed to investigate whether PRKAA1 was a target of miR-181a. Luciferase-mediated luminescence was significantly decreased when HEK293T or HT-22 cells were co-transfected with the miR-181a agomir and the 3′ UTR of PRKAA1 (both *p* < 0.01, Fig. 4a–c). However, the luminescence did not change when the seed regions of PRKAA1 were mutated (both *p* > 0.05, Fig. 4a–c). Thus, miR-181a may directly target PRKAA1.

### miR-181a was involved in the formation of hippocampus-dependent memories by regulating PRKAA1

**Figure 5:**
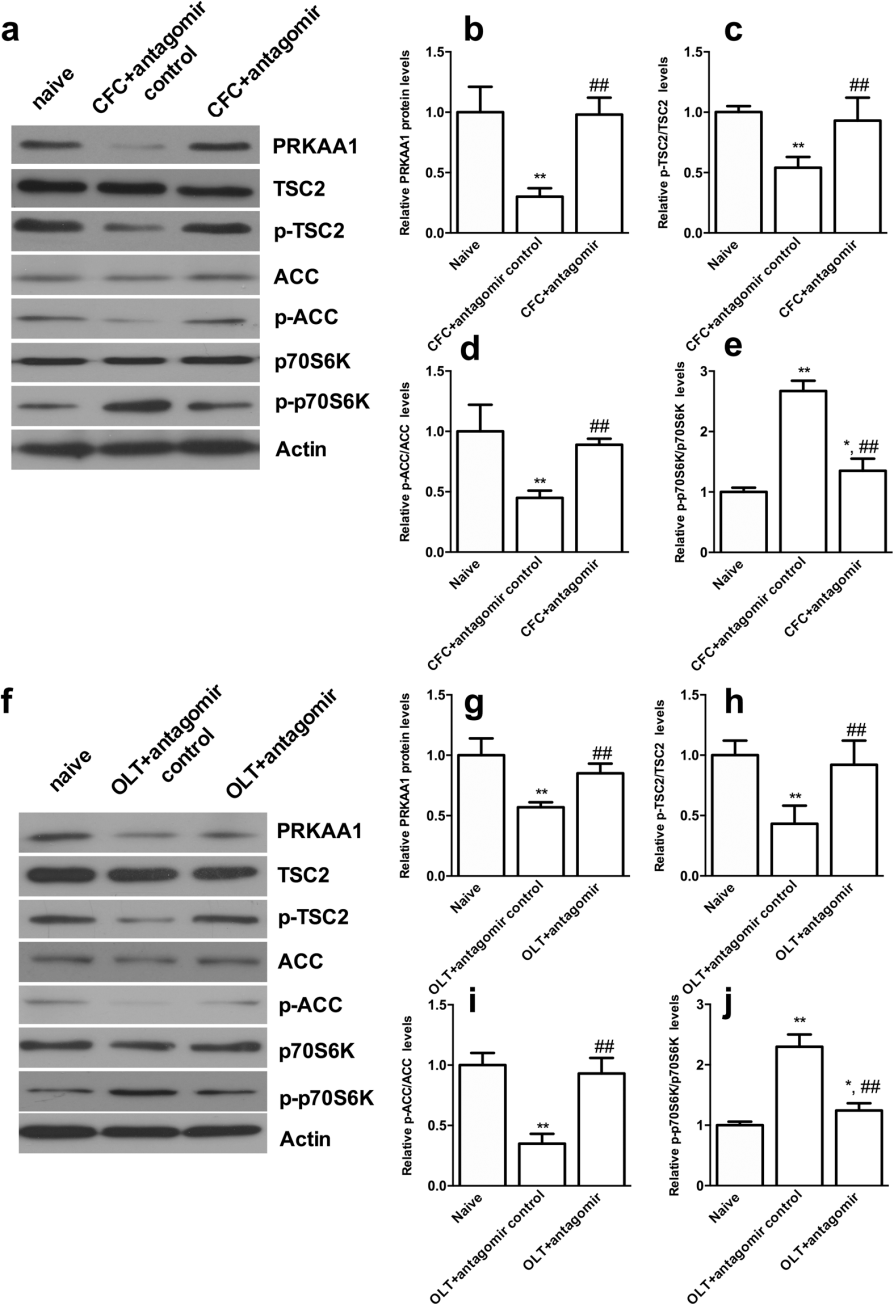
miR-181a was involved in LTM formation following CFC and OLT by regulating the expression and activity of PRKAA1. (**a**) Representative cropped western blots of PRKAA1, TSC2, p-TSC2, ACC, p-ACC, p70S6K and p-p70S6K protein levels in the mouse DH 2 h after CFC training. Original uncropped western blots are provided in a Supplementary Figure 1. (**b,e**) Statistical analysis of the western blot results. *p < 0.05, **p < 0.01, compared with the naïve group. ^##^ *p* < 0.01, compared with the CFC + antagomir control group. (**f**) Representative cropped western blots of PRKAA1, TSC2, p-TSC2, ACC, p-ACC, p70S6K and p-p70S6K protein levels in the mouse DH 2 h after OLT training. Original uncropped western blots are provided in a Supplementary Figure 2. (**b,e**) Statistical analysis of the western blot results. *p < 0.05, **p < 0.01, compared with the naïve group. ^##^ *p* < 0.01, compared with the OLT + antagomir control group. n = 5 per group. All values are presented as means ± SEM.

We measured the levels of the PRKAA1 protein after CFC and OLT training to verify whether endogenous miR-181a regulated PRKAA1 expression during CFC- and OLT-dependent memory formation. Levels of the protein levels of PRKAA1 in the DH decreased significantly 2 h after CFC or OLT training (both *p* < 0.01); this change was effectively blocked by the miR-181a antagomir (both *p* < 0.01, Fig. 5a,b). Moreover, the phosphorylation of downstream targets of PRKAA1, TSC2 and ACC, decreased significantly 2 h after CFC or OLT training (all *p* < 0.01), whereas the miR-181a antagomir treatment reversed these decreases (all *p* < 0.01, Fig. 5c,d for CFC, Fig. 5h,I for OLT). Based on these findings, miR-181a effectively regulated both the expression and activity of PRKAA1 during the formation of hippocampus-dependent memories. Because PRKAA1 is an important regulator of the mTOR signaling pathway, the relationship between miR-181a and the mTOR signaling pathway was also evaluated by monitoring the phosphorylation of the mTOR substrate p70-S6Kinase (p70S6K). CFC or OLT training increased the phosphorylation of p70S6K (both *p* < 0.01), whereas the miR-181a antagomir treatment reversed the changes in p70S6K phosphorylation (both *p* < 0.01, Fig. 5e for CFC, Fig. 5j for OLT). Thus, miR-181a might be involved in the formation of hippocampus-dependent memories by regulating the PRKAA1- mTOR signaling pathway.

**Figure 6:**
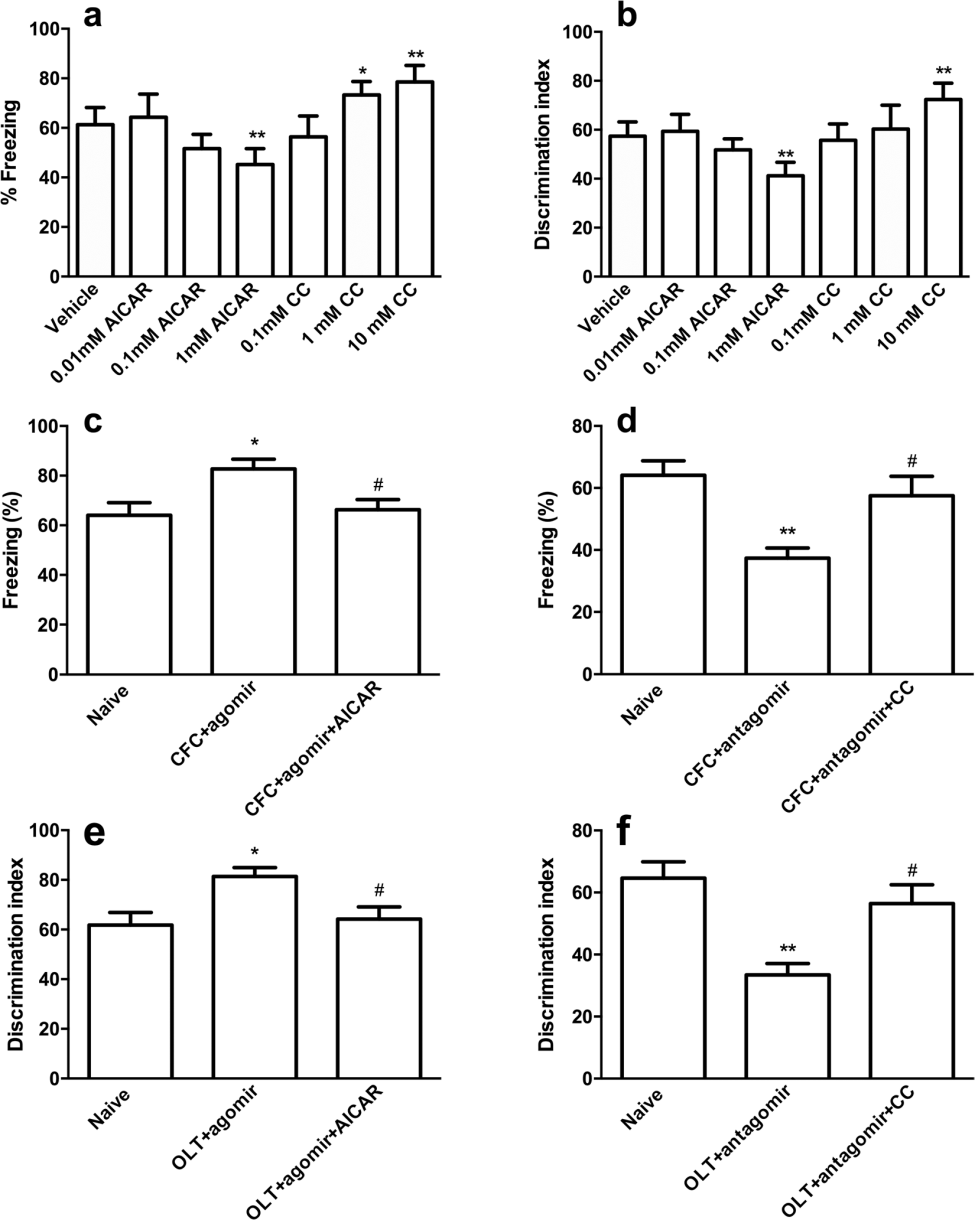
miR-181a was involved in LTM formation following CFC and OLT by regulating PRKAA1. (**a,b**) Effects of different doses of the PRKAA1 agonist and inhibitor on the freezing response (**a**) and discrimination index (**b**) of mice 24 h after CFC or OLT training. *p < 0.05, **p < 0.01, compared with the vehicle group. n = 7 per group. (**c**–**d**) Freezing response when tested 24 h after CFC training. *p < 0.05, **p < 0.01, compared with the naïve group. ^#^ *p* < 0.05, compared with the CFC + miR-181a agomir group or CFC + miR-181a antagomir group. (**e**–**f**) Discrimination index 24 h after OLT training. *p < 0.05, **p < 0.01, compared with the naïve group. ^#^ *p* < 0.05, compared with the OLT + miR-181a agomir group or OLT + miR-181a antagomir group. CC, compound C. n = 8 per group. All values are presented as means ± SEM.

Then, mice were treated with the PRKAA1 agonist AICAR and inhibitor CC to manipulate the kinase activity of PRKAA1 and to further evaluate the role of PRKAA1 in miR-181a regulated CFC- and OLT-dependent memory formation. We examined the effects of different doses of the PRKAA1 agonist (AICAR: 0.01, 0.1 or 1 mM) and inhibitor (CC: 0.1, 1 or 10 mM) on hippocampus-dependent memory formation. The 1 mM AICAR treatment significantly decreased both the freezing time and the discrimination index 24 h after CFC or OLT training (both *p* < 0.01). Conversely, 10 mM CC significantly increased both the freezing time and the discrimination index significantly 24 h after CFC or OLT training (both *p* < 0.01, Fig. 6a,b). Therefore, in the subsequent experiments, 1 mM AICAR and 10 mM CC were used.

When AICAR was microinjected into the DH, the increased freezing times caused by the miR-181a agomir treatment were reversed in the CFC test (*p* < 0.01, Fig. 6c). Conversely, the PRKAA1 inhibitor CC significantly rescued the decreased freezing times observed in the miR-181a antagomir treated mice (*p* < 0.01, Fig. 6d).

In the OLT test, the increased discrimination index observed in the miR-181a agomir-treated mice was decreased by AICAR (*p* < 0.01, Fig. 6e). The PRKAA1 inhibitor CC rescued the decreased discrimination index observed in miR-181a antagomir-treated mice (*p* < 0.01, Fig. 6f).

Thus, PRKAA1 was a direct target of miR-181a and was involved in hippocampus-dependent memory formation.

## Discussion

In this study, miR-181a was involved in hippocampus-dependent memory formation. Using the bioinformatics, luciferase and gain/loss-of-function assay in mice, miR-181a targeted PRKAA1, which mediated the roles of miR-181a in memory formation.

Initially, miR-181a was shown to act as a tumor suppressor that triggered growth inhibition, induced apoptosis and inhibited the invasion of glioma cells^14^. Then, the roles of miR-181a in the proliferation, migration or treatment of many kinds of cancers, including cervical cancer, oral squamous cell carcinoma, breast cancer, gastric cancer, coloreactal cancer, etc, were reported and attracted substantial attention^32–37^. Based on the recent finding that miR-181a is widely distributed in the brain, its roles in physiological and pathological processes in the central nervous system have been explored. According to a few studies, miR-181a is one of the key regulators of the reward circuit in the brain and might be implicated in cocaine addiction^38–40^. miR-181 also influences apoptosis and mitochondrial function in astrocytes and neurons^41, 42^, and protects the brain from cerebral ischemia^43–45^. Moreover, miR-181a may serve as a promising therapeutic target for epilepsy^26, 46^. However, the roles of miR-181a in advanced physiological functions, such as learning and memory, remain unclear. In our study, miR-181a levels increased significantly after CFC or OLT training; moreover, the inhibition or overexpression of miR-181a impaired or enhanced the CFC- and OLT-dependent memory formation, respectively. Our study was the first to show that the tumor suppressor miR-181a was also involved in hippocampus-dependent memory formation. Our study may provide novel insights into the molecular etiology of memory. Certainly, future studies examining the role of miR-181a in other hippocampus-dependent and -independent memory paradigms will help determine the exact roles of this miRNA in different cognitive processes. Interestingly, the increased miR-181a levels observed after OFC and OLT training were transient. Although miRNAs were initially considered highly stable molecules, rapid miRNA degradation has now been reported in various organisms and experimental systems^47^. Strikingly, rapid miRNA degradation appears to be a general property of neurons. As shown in the study by Krol and colleagues, the levels of the miRNA-cluster miR-183/96/182, miR-204 and miR-211 in the retina decreased to approximately 50% of the initial level within 90 min after mice were shifted from light to dark conditions^48^. Moreover, the half- lives of miR-125b, miR-132, miR-146a, mir-183, miR-9 in primary human neuron cultures and postmortem human brain tissues were no longer than 3.5 h^29^. Notably, the fast turnover of neuronal miRNAs might be related to neuronal activity. The use of tetrodotoxin to block action potentials or the inhibition of glutamate receptors prevented fast turnover^48^. Therefore, we reasonably postulated that miR-181a exhibited rapid turnover after CFC or OLT training, but the underlying mechanisms must be studied in the future.

In addition, PRKAA1 was one of the target genes of miR-181a in this study. The protein encoded by PRKAA1 is the catalytic subunit of AMPK, which belongs to the ser/thr protein kinase family. AMPK is an energy sensor that is activated by stimuli that increase the cellular AMP/ATP ratio. A number of key metabolic enzymes are activated by AMPK through phosphorylation and are subsequently involved in many biological functions. After CFC or OLT training, the expression and activity of PRKAA1 decreased according to the increase in miR-181a expression, and the activation of AMPK in the DH could reverse the effects of miR-181a on the LTM formation following CFC and OLT. However, AMPK inhibition in the DH had the opposite effects. These results were consistent with previous reports showing that AICAR impairs L-LTP, whereas CC could improves L-LTP^31^. It might explain the underlying mechanism by which miR-181a regulates the formation of CFC- or OLT-dependent memories. Certainly, direct evidence for the roles of miR-181a in L-LTP would be more convincing, and this study will be performed in the future. Moreover, this study is the first to indicate that PRKAA1 encoded AMPK was one of the most important targets of miR-181a during hippocampus-dependent memory formation. AMPKα1 has been shown to inhibit mammalian target of rapamycin (mTOR) by activating of the TSC1/TSC2 complex and subsequently inhibiting mTOR with the help of phosphorylated TSC2 (21). mTOR is a kinase complex that phosphorylates and activates some proteins, including p70S6K kinase, which phosphorylates the downstream target ribosomal protein S6 (rpS6)^50, 51^. As shown in our study, p70S6K kinase was activated after CFC or OLT training following miR-181a regulation. These downstream effectors act to increase the translation of specific mRNAs, most of which are related to the various forms of synaptic plasticity underlying the cognitive functions in the brain. Therefore, the effects of miR-181a on the formation of long-term memories may be related to the AMPK-regulated mTOR signaling pathway.

In conclusion, this study was the first to show that miR-181a participated in hippocampus-dependent memory formation via regulating the expression and activity of PRKAA1. Our study might shed light on the regulation of cognition and future treatments for cognitive disorders.

## Electronic supplementary material

Supplementary figure
