# Supplementary material for: miR-181a involves in the hippocampus-dependent memory formation via targeting PRKAA1
Source: Sci Rep. 2017 Aug 16;7:8480. doi: 10.1038/s41598-017-09095-3 (PMC5559581; doi:10.1038/s41598-017-09095-3)
Supplement: Supplementary file 1 — Supplementary figure [file 41598_2017_9095_MOESM1_ESM.pdf]

miR-181a involves in the hippocampus-dependent memory formation via targeting PRKAA1

Sun-fu Zhang[1,2*], Jun-chen Chen[3*], Jing Zhang[1], Jian-guo Xu[1#]

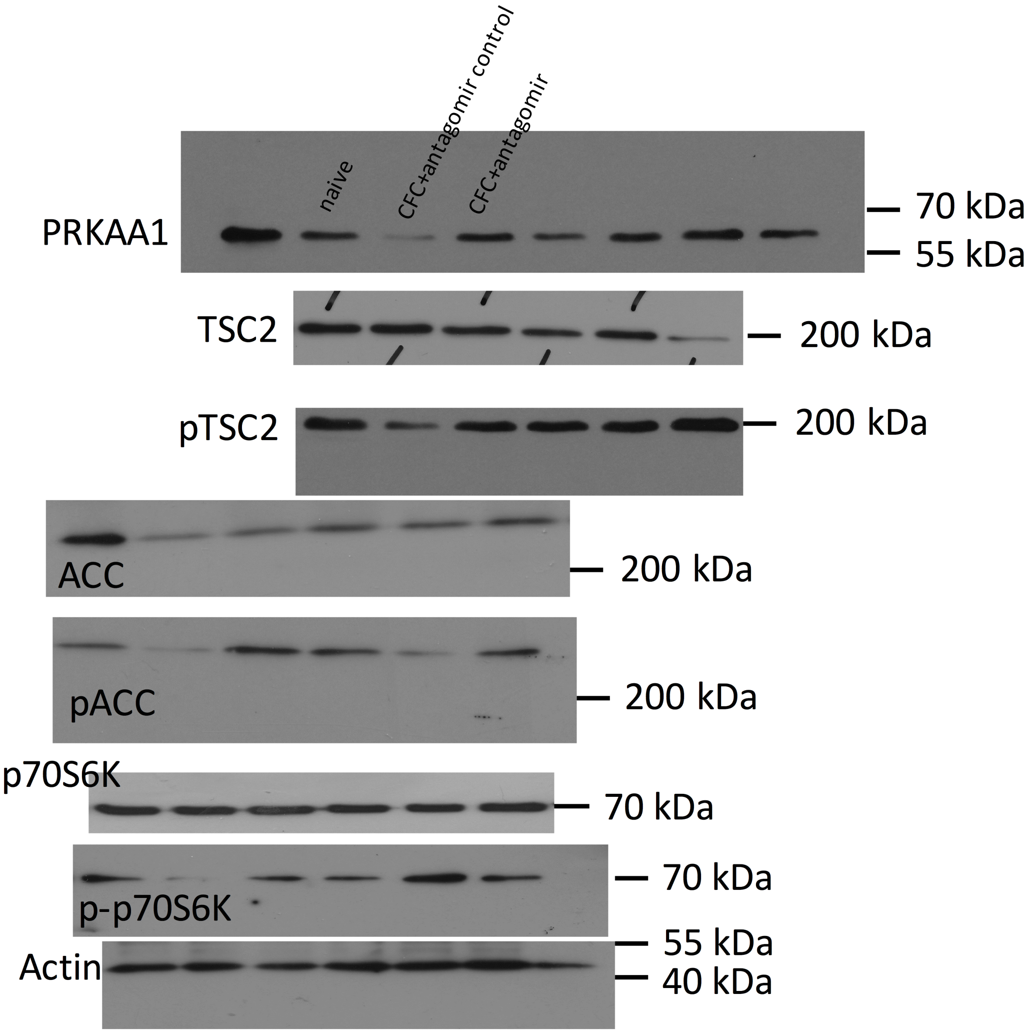

Supplementary Figure 1. Original uncropped Western blots of the images reported in Fig. 5a.

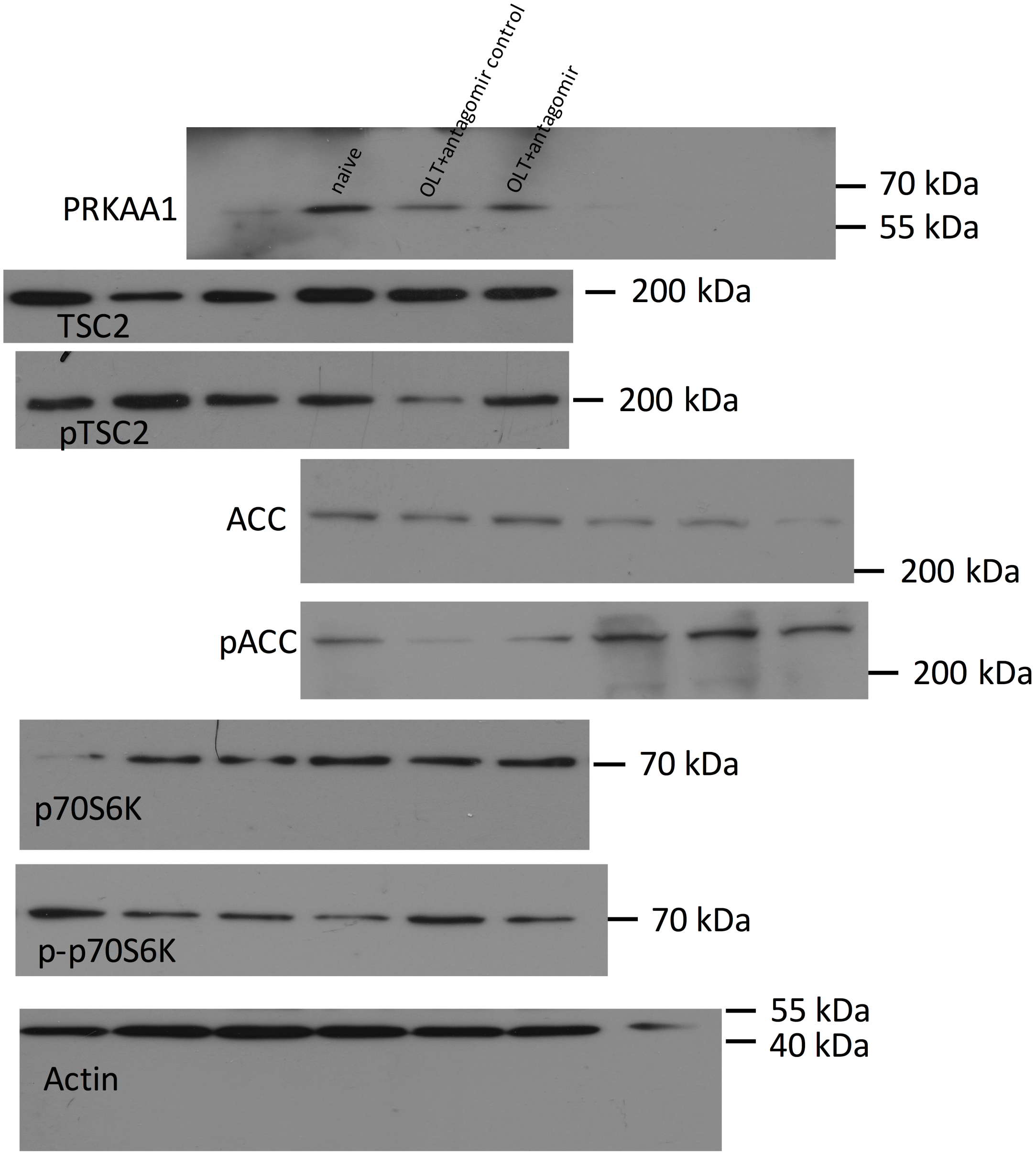

Supplementary Figure 2. Original uncropped Western blots of the images reported in Fig. 5f.
